# Facts Concerning the Noxiousness of Rain-Water and Dew in 1797: Communicated in a Letter from Dr. Foot to Dr. Miller, of Greensburg, N. America

**Published:** 1808-06

**Authors:** 


### FACTS *concerning the* NOXIOUSNESS *of* RAIN-WATER *and* DEW *in* 1797: *Communicated in a Letter from Dr.* FOOT *to Dr.* MILLER, *of Greensburg, N. America.*

IT is with pleasure that I comply with your request, to furnish you with the facts concerning the morbid effects of rain-water in this quarter of the country, in the summer of 1797. As I was not a resident within this State at that time, and, of course, not a personal observer, I have delayed compliance until I could inform myself of more particulars, of the extent, duration, and other circumstances of the occurrence.

After making particular inquiry, I find but few persons of common curiosity, of those who had been in a condition for this kind of observation, who had not noticed these uncommon appearances; and when I consider the publicity and great singularity of the facts, conjoined with their importance, in support of certain physical principles, and the intense thirst for philosophical researches, which characterizes the present age, I have been surprised that they have not heretofore found way to your Repository; a work which I cannot but consider as the proper receptacle for every thing novel or interesting in the philosophical world, and which, for the interests of medicine, it is to be regretted, had not been able to boast of an earlier origin in this country. Facts, of sufficient importance, from which to infer new principles, or even to support analogies, are not of daily occurrence, and may not be repeated in the same country in so interesting a manner in an age: considerations which render a work like yours essentially important, and which ought to operate as a powerful stimulus to country practitioners of medicine within the United States, whose sphere of observation, it must be acknowledged, is comparatively great.

Since the late improvements in Chemical science, and the writings of Dr. DARWIN (who, however, in some of his his principles, was anticipated by the ingenious Brown), I believe, that the identity of those laws which govern animal and vegetable bodies is pretty generally acknowledged. This is a discovery of incalculable importance to the interests of medicine. It has presented to the physician an extensive field for improvement by analogical reasoning, and has called to the aid of medicine almost every other science. For amusement, the physician stands on a level with the Botanist and Chemist; and, in short, the healing art, instead of a mere collection of symptoms and prescriptions, incoherent, and frequently contradictory, has become reducible to calculation, and merits the title of a science, affording the ingenious student a field for amusement, as engaging as it is extensive.

Craving your indulgence for the preceding, and I had almost said involuntary observations, the facts of which I promised you a relation were as follows:—The latter part of the summer of 1797, was rendered remarkable, by the fall of an uncommon quantity of rain. It commenced about the 15th or 16th of July, and continued, with short intermissions, until past the middle of the succeeding month. The earth, by this means, became so loaded with water, that it was almost impossible to walk with dry feet, even over the most elevated fields. The consequence was, in this quarter of the country, a great loss of property experienced in the damaging of much hay, and in the prostration of some valuable mill-dams, with their mills.

But the greatest singularity attending this season was manifested in the inconvenience resulting from the effects of this water on the bodies of that class of people (farmers) who were necessarily most exposed to its influence. This appeared in an uncommon and troublesome soreness of the hands and feet of those, particularly, who were occupied in saving their hay from the rain in low-land meadows. An erythematous inflammation of the whole part immersed in water, frequently took place on the same day; and, in addition to this, often a complete abrasion of the cuticle of the whole foot and leg, in many attended with such a degree of pain and tumefaction as to lay the subject by. In others, besides the above effect, there frequently arose on the part, more especially between and about the toes, where the water would be longest retained, a livid-coloured vesicle, which, on breaking, discharged a dark grumous-like matter; this left behind an ulcer of the phagedænic species, which became difficult to heal, and continued troublesome even for weeks. But the most striking striking proof of acrimony in this water was, that a swelling and soreness (as I am credibly informed) took place, in some instances, on the same day, after slightly wetting the feet, in walking through the dewy grass of uplands in the morning. These appearances were generally noticed as singular; yet few suspected them the result of any morbid quality in the water; and as one of the most striking (though not less mischievous) propensities of human nature is, that in case the real causes of effects do not readily present, to impute them, however singular, to the most obvious; so the conjectures on this occasion were various, vague, and very unsatisfactory. Whether any singularity in this water was discernible by the organs of taste, I have not been able certainly to learn. On asking an old gentleman, a neighbour of mine, whether he tasted of this water, he replied, with a degree of interest, "that, when at labour from his house he had occasion to take a draught from a small rill issuing from the ground, it affected him so sensibly, that he did not forget it in a number of days." Instances of this kind may have been more frequent, but as the quality of water is materially changed by filtration through the earth, it is probable they were not numerous.

I have not likewise been able to satisfy myself, whether the health of other animals was materially affected; certain, however, it is, that swine, at this time, and often, were attacked with a disease, which proved, among them, unusually mortal. The symptoms were, a loss of appetite, and consequent emaciation, attended with a discharge from the inner angle of the eye of a frothy limpid fluid, producing a depilation, and even excoriation of the whole cheek in its course. To what cause this singular complaint among that species of animals is imputable I shall not conjecture. The remedies which proved of most efficacy in saving them, I am informed, was a free exhibition of brimstone combined with their food. If such were the effects of these waters on animal bodies, we should expect them to be at least not less conspicuous on vegetables: these appeared particularly in the destruction of tender garden plants, such as cucumbers, &c. and in a discolouration of the grass where the water remained any time, and in its complete destruction in patches about meadows, as effectually as if scorched by fire.

I have been more particular in a relation of these appearances, as they took place some time past. With respect to their extent, I believe, from the best information I have been able to obtain, that they were limited by the south south of this, but extended northwardly quite into Dutchess county, easterly to the distance of a few miles only. The circumstance of limitation seems to involve some obscurity. Neither have I been able to learn at what period of this rainy season these effects were most conspicuous.

I am not qualified to hazard any conjecture on this subject. You will recollect that the summer of 1797 was rendered remarkable by numerous occurrences, which go strongly to establish the doctrine of ocult causes in the atmosphere—a doctrine which contains many valuable arcana, and from a developement of which important acquisitions will result to the science of medicine.

Did these waters possess an acrimonious quality previously to their precipitation? If so, was it of volcanic origin? Was the atmosphere of that season compounded of more than its usual quantity of azote? Or did the morbid properties of this water arise from its meeting with some agent on the surface of the earth? If so, was this septic acid simply from vegetable decomposition, aided by the water as a menstruum? And did this water possess any unusual quality which favoured this decomposition?

